# Psoriasis caused by camrelizumab in a patient with esophageal cancer: a case report

**DOI:** 10.3389/fmed.2025.1647297

**Published:** 2025-10-02

**Authors:** Yimei Duan, Jie Chen, Dongsheng Zhong, Xincai Xiong

**Affiliations:** Affiliated Hospital of North Sichuan Medical College, Nanchong, Sichuan, China

**Keywords:** camrelizumab, psoriasis, immune-related adverse effects, PD-1 inhibitor, esophageal cancer

## Abstract

Immune checkpoint inhibitors (ICIs) are an emerging treatment strategy for cancer, working by activating T cells to suppress tumor growth. However, they can cause immune-related adverse events (irAEs), including psoriasis. We report a case of a patient with esophageal cancer who developed psoriasis 6 weeks after starting camrelizumab. The condition improved following the discontinuation of camrelizumab and treatment with a topical glucocorticoid and a vitamin D3 derivative ointment. At 6-month follow-up, the patient showed no recurrence of psoriatic lesions or tumor progression.

## Introduction

In recent years, immune checkpoint inhibitors (ICIs) have emerged as a major strategy for treating malignant tumors by enhancing T-cell-mediated tumor suppression. Commonly used ICIs include ibritumomab, ravulizumab, and sindilizumab. Psoriasis is one of the recognized immune-related adverse event (irAEs) associated with ICI use. According to existing reports, most cases of ICI-induced psoriasis have been linked to ravulizumab or pembrolizumab, with only one previously reported case associated with camrelizumab (1). This paper presents a case of camrelizumab-induced psoriasis in a patient with esophageal cancer, where the skin lesions significantly improved following drug discontinuation and treatment with oral glycyrrhizin tablets, topical glucocorticoids, and a vitamin D3-derived ointment.

## Case report

The patient was a 73-year-old man who presented to the hospital with a 2-month history of widespread erythema, papules, plaques, scaling, and pruritus. He had undergone surgery for esophageal cancer and received camrelizumab injections (200 mg every 20 days) prior to symptom onset. Approximately 6 weeks after receiving the first dose, or approximately 2 days after receiving the second dose, the patient developed diffuse erythema, scaling, and plaques with severe itching, which progressively worsened. Initial treatment at a local hospital was ineffective, and the patient was referred to our facility.

He had no previous history of psoriasis, food or drug allergies, special personal history, or a similar medical history in the family. The patient denied a history of metabolic diseases such as diabetes and thyroid dysfunction.

### Physical examination

**Figure 1:**
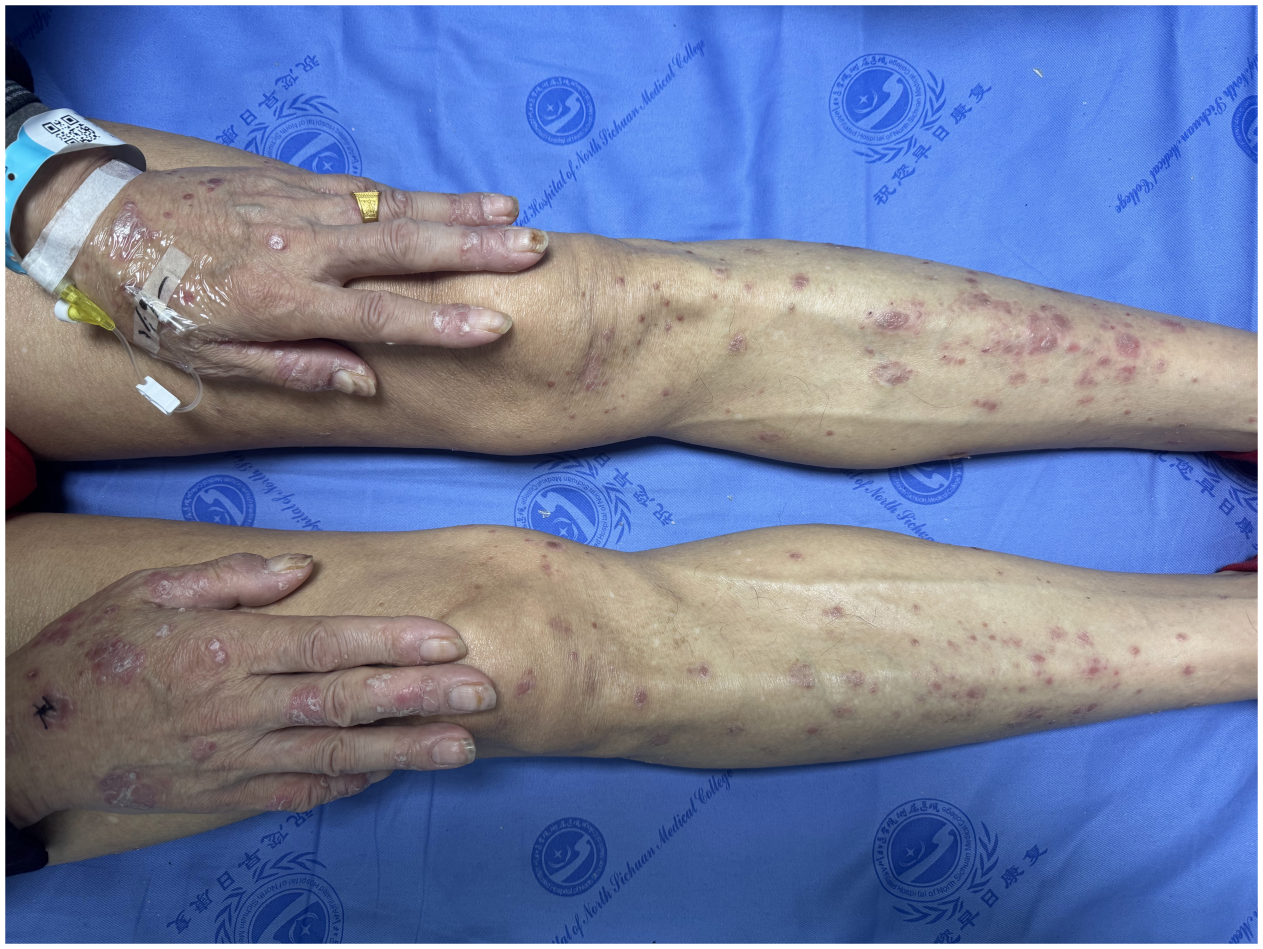
Dermatological examination revealed widespread dark red patches, papules, and plaques covered with white scales.

Systematic examination was unremarkable. Dermatological examination revealed widespread dark red patches, papules, and plaques covered with white scales (Figure 1).

### Dermoscopic examination

**Figure 2:**
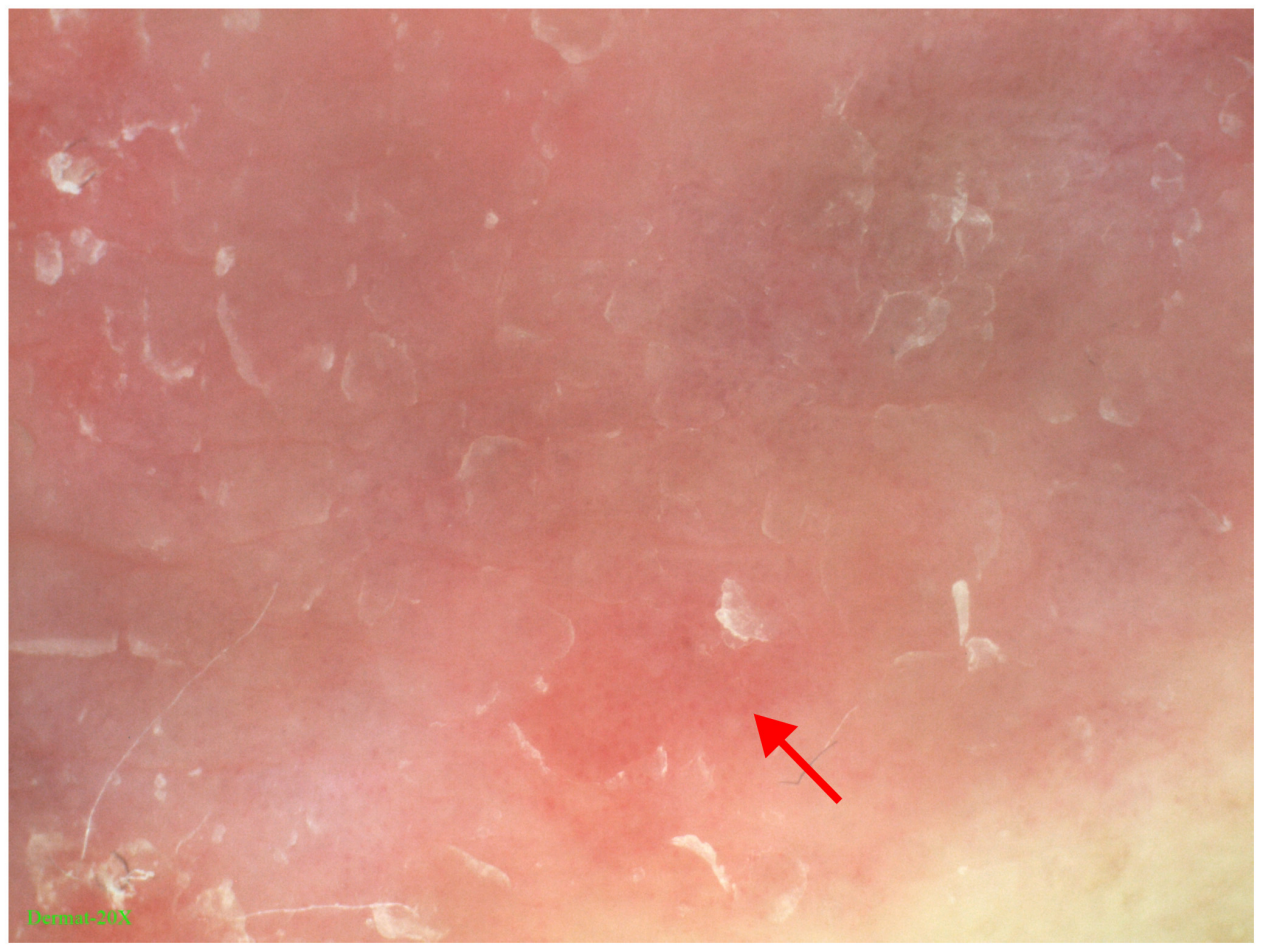
Dermoscopy showed well-demarcated red plaques on a bright red background with evenly distributed red punctate blood vessels and scattered white scales.

Dermoscopy showed well-demarcated red plaques on a bright red background with evenly distributed red punctate blood vessels and scattered white scales (Figure 2).

### Confocal microscopy

**Figure 3:**
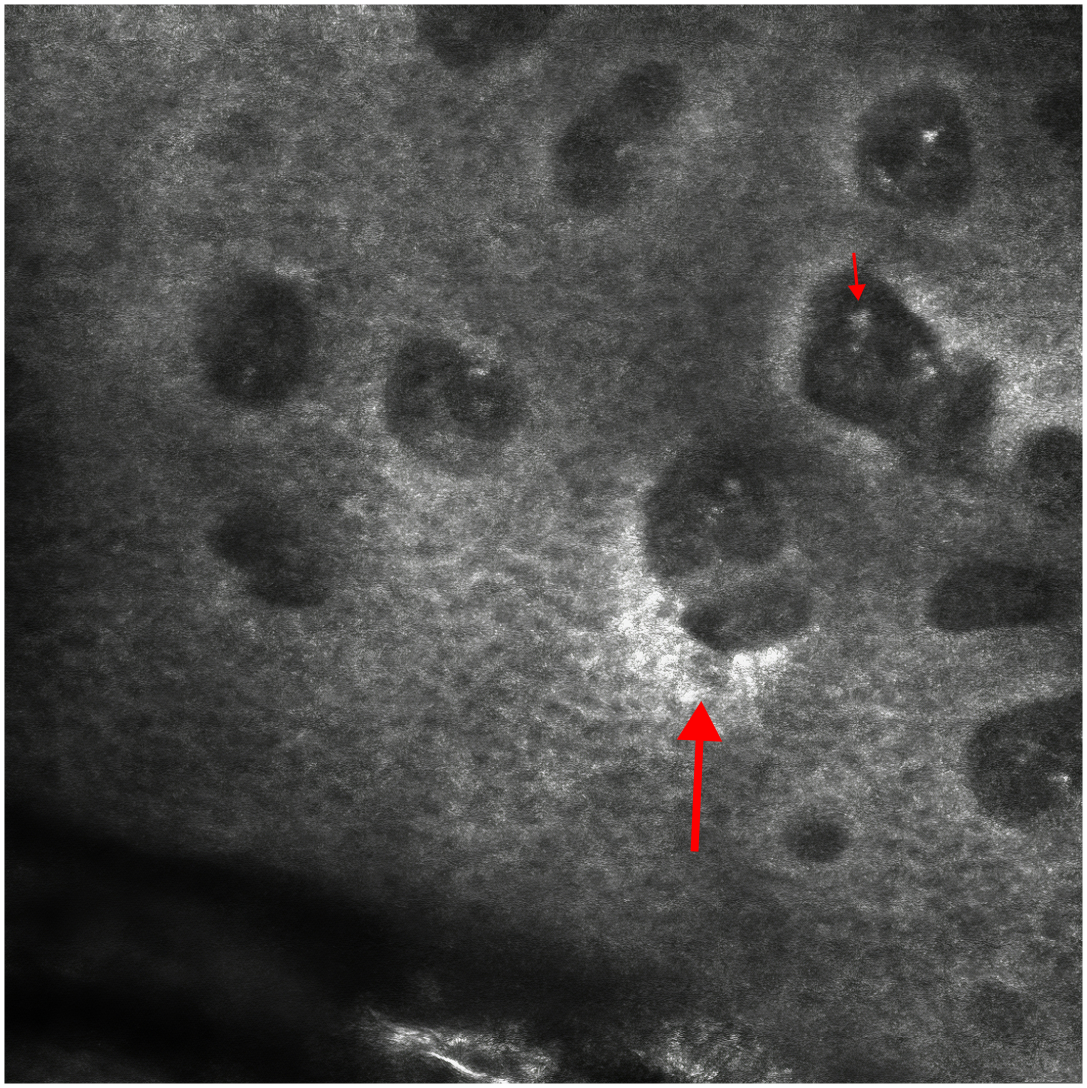
Reflective confocal microscopy of lower limb skin lesions showed elongated dermal papillae, tortuous and dilated capillaries, and perivascular inflammatory cell infiltration.

Reflective confocal microscopy of lower limb skin lesions showed elongated dermal papillae, tortuous and dilated capillaries, and perivascular inflammatory cell infiltration (Figure 3).

### Laboratory examination

Routine blood tests, coagulation profile, HIV antibodies, hepatitis B virus, and syphilis serology tests did not show any abnormalities.

### Histopathological examination

**Figure 4:**
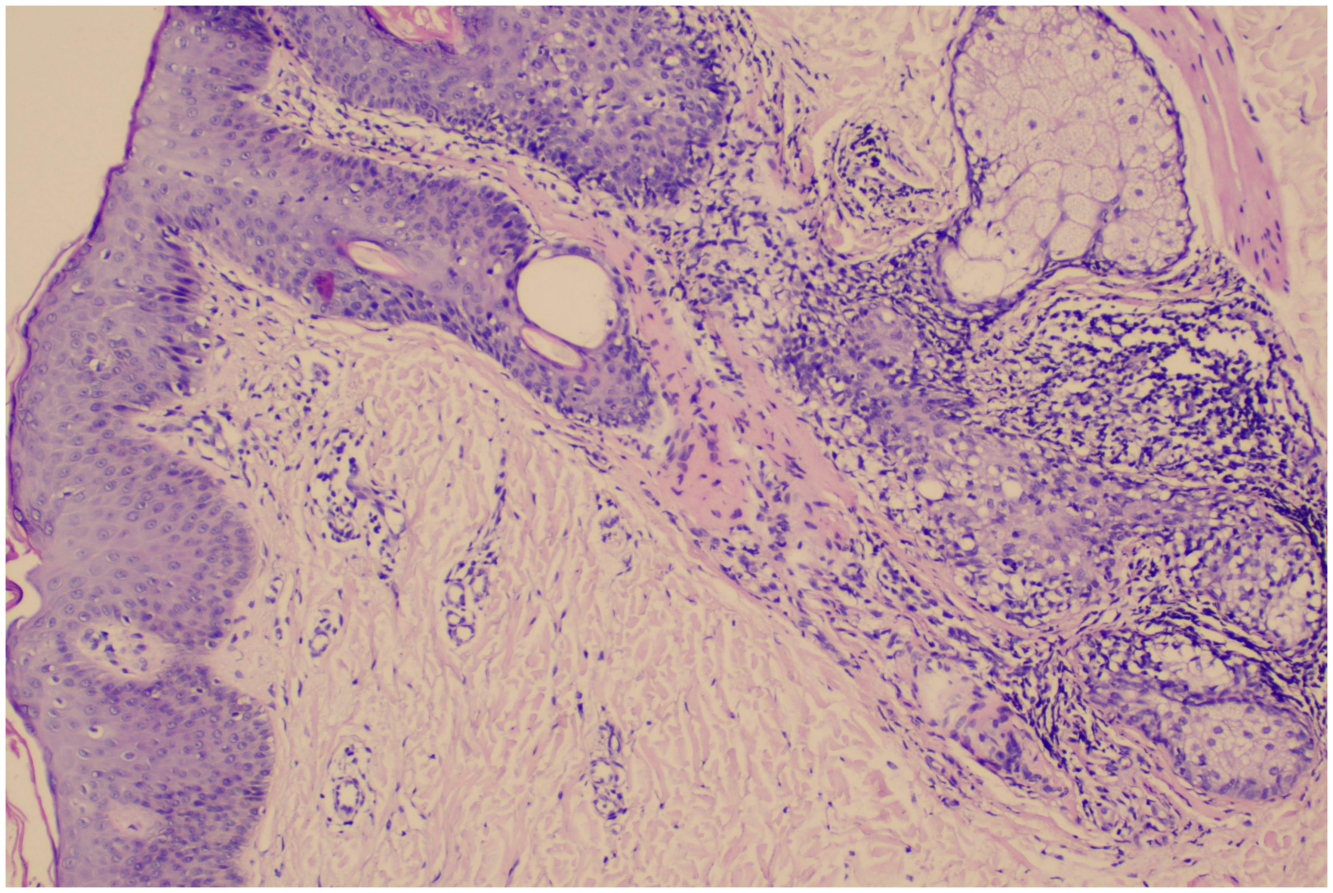
Biopsy of the skin lesions showed epidermal hyperkeratosis with parakeratosis, neutrophil aggregates, and hypertrophy of the stratum spinosum; elongation of dermal papillae, capillary dilatation, and perivascular infiltration by lymphocytes and neutrophils.

Biopsy of the skin lesions showed epidermal hyperkeratosis with parakeratosis, neutrophil aggregates, and hypertrophy of the stratum spinosum; elongation of dermal papillae, capillary dilatation, and perivascular infiltration by lymphocytes and neutrophils (Figure 4).

### Diagnosis

Based on clinical presentation and histopathological findings, the patient was diagnosed with plaque psoriasis. According to the Common Criteria for Adverse Event Evaluation (CTCAE v5.0) classification, patients should be classified as grade 3 (severe), indicating severe symptoms.

### Treatment

Camrelizumab was immediately discontinued. The patient was treated with oral glycyrrhizin tablets, and topical hydrocortisone butyrate and vitamin D3 derivative ointment applied to local lesions twice daily. After treatment, the lesions began to resolve, with noticeable reduction in scaling and erythema within 1 month. Two months later, in a follow-up phone call, the patient had only a few residual skin lesions on the abdomen and was instructed to continue the above medications. By the 6-month follow-up, the patient reported complete cessation of all medications by the third month and no recurrence of skin lesions thereafter. The clinical course strongly suggested camrelizumab as the trigger for the psoriasis flare.

## Discussion

ICIs are a novel class of antitumor drugs widely used to treat advanced malignancies by activating the immune system and enhancing tumor cell clearance. Camrelizumab, a programmed death receptor-1 (PD-1) inhibitor developed in China and approved for marketing in 2019, has become a first-line standard therapeutic option for advanced esophageal cancer. Although ICIs exhibit significant efficacy in antitumor therapy, they may induce multisystem irAEs, including maculopapular rash, herpetic pemphigoid, psoriasis, vitiligo-like hypopigmentation, alopecia, and in severe cases, Stevens-Johnson syndrome/toxic epidermal necrolysis (SJS/TEN)-like manifestations (2, 3). Studies indicate that approximately 0.5% of patients treated with ICIs develop psoriasis, of which 70.8% represent exacerbations of pre-existing disease (4, 5). In a large cohort, Phillips et al. (6) reported a median onset time of 61 days for ICI-related skin rashes. A European multicenter study found that the minimum time required for ICIs to trigger psoriasis was 12 weeks (5). Notably, literature reports suggest that the time to onset of camrelizumab-associated psoriasis is generally shorter than with other ICIs, potentially due to its shorter half-life, although this requires further investigation.

Psoriasis is a common chronic papulosquamous skin disorder, characterized by abnormal immune system activation, with IL-17 and IL-23 considered key pathogenic mediators (7, 8). While the precise mechanism by which PD-1 inhibitors induce psoriasis remains unclear, it may involve immune overactivation (9, 10). This involves the activation of various cell populations, such as neutrophils, dendritic cells, Th1 and Th7 cells, and Treg cells (11). Mild psoriasis induced by ICIs can often be effectively managed with topical glucocorticoids without discontinuing immunotherapy. However, in moderate-to-severe cases, clinicians must balance the need for continued cancer therapy against the severity of skin involvement. Systemic glucocorticoids, although temporarily effective, have limited long-term efficacy and are prone to relapse upon discontinuation (12); therefore they are not recommended as first-line therapy. Avacopan has demonstrated favorable safety and efficacy in the treatment of psoriasis (13). In refractory cases, IL-6 receptor inhibitors may provide significant improvement (14). Therefore, in patients with severe disease, treatment with biologics should be considered after a comprehensive evaluation. In our patient, psoriasis was significantly alleviated with a combination of topical glucocorticoid and a vitamin D3 derivative ointment, along with the discontinuation of camrelizumab. During follow-up, the patient's tumor remained stable, and no recurrence of psoriasis was observed.

A critical question for clinicians is whether to resume ICI therapy after resolution of psoriasis. The safety of reinitiating ICIs and their continued benefit in tumor control must be carefully weighed. An analysis of two retrospective studies revealed that the overall incidence of irAEs after ICIs rechallenge was 52%−55%, comparable to that seen during initial therapy (15, 16). When reinitiation is necessary, switching to an alternative ICI with a different mechanism—such as from anti-CTLA-4 to anti-PD-1/PD-L1 antibodies—may improve safety in eligible patients.

Although the widespread use of ICIs has brought significant benefits to patients with tumors, their associated cutaneous adverse effects can severely impact quality of life, and in some cases, become life-threatening. Clinicians should remain vigilant for potential skin-related irAEs during ICI treatment, ensuring early identification and timely intervention to maintain both treatment efficacy and patient safety.

## Data Availability

The original contributions presented in the study are included in the article/supplementary material, further inquiries can be directed to the corresponding author.
